# Apolipoprotein E Genetic Variation and Its Association With Cognitive Function in Rural-Dwelling Older South Africans

**DOI:** 10.3389/fgene.2021.689756

**Published:** 2021-10-14

**Authors:** Cassandra C. Soo, Meagan T. Farrell, Stephen Tollman, Lisa Berkman, Almut Nebel, Michèle Ramsay

**Affiliations:** ^1^Sydney Brenner Institute for Molecular Bioscience, Faculty of Health Sciences, University of the Witwatersrand, Johannesburg, South Africa; ^2^Division of Human Genetics, National Health Laboratory Service and School of Pathology, Faculty of Health Sciences, University of the Witwatersrand, Johannesburg, South Africa; ^3^Harvard Center for Population and Development Studies, Harvard University, Cambridge, MA, United States; ^4^MRC/Wits Rural Public Health and Health Transitions Research Unit, School of Public Health, Faculty of Health Sciences, University of the Witwatersrand, Johannesburg, South Africa; ^5^Department of Social and Behavioral Sciences, Harvard T.H. Chan School of Public Health, Boston, MA, United States; ^6^Institute of Clinical Molecular Biology, Kiel University, Kiel, Germany

**Keywords:** apolipoprotein E 𝝴4, cognitive function, African population, educational attainment, memory, executive function, visuospatial ability, language

## Abstract

Apolipoprotein E (*APOE*) 𝜀4 allele carrier status is well known for its association with an increased likelihood of developing Alzheimer’s disease, but its independent role in cognitive function is unclear. *APOE* genetic variation is understudied in African populations; hence, this cross-sectional study in a rural South African community examined allele and genotype frequencies, and their associations with cognitive function. Cognitive function was assessed using two different screening methods to produce a total cognition score and four domain-specific cognition scores for verbal episodic memory, executive function, language, and visuospatial ability. Cognitive phenotype and *APOE* genotype data were used to determine whether *APOE* variation was significantly associated with cognitive function in this population. Observed allele frequencies for 1776 participants from the HAALSI study [age 40–80years (mean=56.19); 58.2% female] were 58.1% (𝜀3), 25.4% (𝜀4) and 16.5% (𝜀2). Allele distributions were similar to the African super population, but different from all non-African super populations from the 1,000 Genomes Project. The 𝜀3 homozygous genotype was most common (34.9%) and used as the base genotype for comparison in regression models. Four models were tested for each of the five cognitive phenotypes to explore association of *APOE* variation with cognitive function. In the first model assessing association with all genotypes for all individuals, marginally significant associations were observed for 𝜀2 homozygotes where executive function scored higher by ~0.5 standard deviations (*p*=0.037, SE=0.23), and for 𝜀3/𝜀4 heterozygotes where visuospatial ability scores were lower (*p*=0.046, SE=0.14). These did not survive correction for multiple testing. Regional African population differences were observed at the *APOE* locus. Marginally, significant associations between *APOE* genotype, and executive function and visuospatial ability indicate the need for larger studies to better examine these associations in African populations. Furthermore, longitudinal data could shed light on *APOE* genetic association with rate of change, or decline, in cognitive function.

## Introduction

Studies conducted in different populations (mostly of European ancestry) have reported associations between apolipoprotein E (*APOE*) 𝜀4 and various detrimental outcomes. These include susceptibility to, and severity of cardiovascular diseases, HIV infection and its associated comorbidities, late onset Alzheimer’s disease, and recently COVID-19 (Hendrie et al., 1995; Savitz et al., 2006; Burt et al., 2008; Chang et al., 2011; Panos et al., 2013; Kotze et al., 2015; O’Donoghue et al., 2018; Kuo et al., 2020). Aside from its well-known involvement in disease pathogenesis, there is interest in understanding the role of *APOE* variation in normal cognitive function throughout lifespan. This is especially true as studies begin to identify associations across the globe in heterogeneous populations (Kotze et al., 2015). Furthermore, cognition is a complex multifactorial trait and studies examining normal cognitive ageing and brain morphology have observed associations with *APOE* (Small et al., 2004; Wisdom et al., 2011; Rebeck, 2017; O’Donoghue et al., 2018; Hays et al., 2019; Reas et al., 2019). These results have, however, been inconsistent, and larger meta-analyses have observed small, significant differences in cognitive performance and brain morphology in European populations between 𝜀4-carriers and non-𝜀4-carriers (Small et al., 2004; Wisdom et al., 2011; O’Donoghue et al., 2018). Studies focused on Alzheimer’s disease (AD) have shown that the presence of 𝜀4 is associated not only with increased susceptibility to AD, from 2-fold to 33-fold depending on the number of risk alleles and ethnicity, but also with an earlier age of onset of the disease (Wisdom et al., 2011; Liu et al., 2013; Wendelken et al., 2016; Rebeck, 2017). It has also been found to be associated with other neurocognitive phenotypes, including mild cognitive impairment (MCI), HIV-associated neurocognitive disorder (HAND), brain atrophy and structural pathologies, and the pace of age-related cognitive decline (Burt et al., 2008; Chang et al., 2015; Hendrie et al., 2015; Wendelken et al., 2016; Lipnicki et al., 2017; Espinosa et al., 2018; O’Donoghue et al., 2018; Reas et al., 2019). However, the results of these studies were mixed, as were the strengths of associations and effects for different ethnic groups (Wisdom et al., 2011; Panos et al., 2013; Lipnicki et al., 2019; Reas et al., 2019). This may be due to differences in study design, sample size, characterisation and collection of cognition data, and genetic diversity due to differences in ethnicity.

The *APOE* alleles 𝜀2, 𝜀3, and 𝜀4 encode structurally different protein isoforms with variable molecule-binding affinity (Corbo and Scacchp, 1999). APOE is involved in lipid metabolism, specifically of cholesterol, and is integral to cholesterol transport and clearance within the central nervous system (Rebeck, 2017). Global variation in *APOE* allele frequencies have been reported 185 between ethnic groups (Corbo and Scacchp, 1999; Demarchi et al. 2005; Singh et al., 2006. The 𝜀3 allele is the most common allele across all populations, and 𝜀4 frequencies are higher in African and aboriginal populations than in Europeans and Asians (Sandholzer et al., 1995; Corbo and Scacchp, 1999; Singh et al., 2006; Liu et al., 2013). Despite this, evidence for its association with AD and other neurocognitive phenotypes in populations of African ancestry is either lacking, or very weak (Osuntokun et al., 1995; Willis et al., 2003; Gureje et al., 2006; Chen et al., 2010; Liu et al., 2013; Morgan et al., 2013; Chang et al., 2015; Hendrie et al., 2015).

Genomic studies of cognitive function among Africans are limited by small sample sizes and low transferability of cognitive performance tests to regions where access to formal education and lower levels of literacy may affect reproducibility of results observed in largely European cohorts (Savitz et al., 2006; Humphreys et al., 2017; Kobayashi et al., 2019; Farrell et al., 2020). The Health and Aging in Africa: A Longitudinal Study of an INDEPTH Community in South Africa (HAALSI) was established to assess trends in sociodemographic determinants, morbidity, and mortality associated with population greying and the current epidemiological and behavioural transition underway in South Africa (SA) (Gómez-Olivé et al., 2018; Houle et al., 2019). Cognitive performance data were captured for over 5,000 individuals aged 40years and older from rural communities in Bushbuckridge, Mpumalanga, SA, a region known to have a high prevalence of HIV (Gómez-Olivé et al., 2018; Asiimwe et al., 2020; Rosenberg et al., 2020). Two screening tools were used to capture cognitive function: (1) a culturally adapted version of the United States Health and Retirement Study (US HRS) screening tool translated into the local language Shangaan and (2) the tablet-based Oxford Cognition Screen (OCS-Plus), which was adapted and validated in this study population (Humphreys et al., 2017; Gómez-Olivé et al., 2018; Kobayashi et al., 2019; Farrell et al., 2020). It provides domain-specific scores for cognitive function in episodic memory, executive function, language, and visuospatial ability without bias to levels of literacy or numeracy (Humphreys et al., 2017; Kobayashi et al., 2019; Farrell et al., 2020). Here, a subset (*n*=1776) of the larger HAALSI cohort was genotyped for the *APOE* locus. Our aim was to examine the associations of *APOE* with total cognition score and specific cognitive domains adjusting for known moderators, such as age, level of education and sex. HIV status was also included as a covariate, and we sought to explore a possible relationship between HIV status and *APOE* in this cohort.

## Materials and Methods

### Study Design and Participants

Demographic, socioeconomic, health and cognitive function data were collected from 5,059 adults (men and women, aged 40years and older) recruited from Bushbuckridge, rural Mpumalanga, South Africa, from November 2014 to November 2015, as the baseline for the HAALSI longitudinal study (Gómez-Olivé et al., 2018). African participants who had been living in the area for at least 12months preceding data collection were selected from the Health and Demographic Surveillance System database and consented (Kahn et al., 2012). Cognitive function variables: HIV status, age, sex, and educational attainment expressed as level of education (none, primary, high and tertiary) were used in this population-based cross-sectional community study. Sample size differed per outcome due to missing data. Approximately 2000 of these same participants overlapped with the Africa Wits-INDEPTH Partnership for Genomic Studies (AWI-Gen) for which blood samples were collected for DNA extraction and genetic analyses (Ramsay et al., 2016; Ali et al., 2018). Data collection and informed consent templates for the HAALSI and AWI-Gen studies were approved by the University of the Witwatersrand, Johannesburg, Human Research Ethics Committee [Wits HREC (Medical); M141159, M121029], the Harvard T.H. Chan School of Public Health, Office of Human Research Administration (C13-1608-02), and the Mpumalanga Provincial Research and Ethics Committee (approved: 2014/10/22). Ethics approval for this study was independently granted by Wits HREC (Medical) (M170916).

### Cognition Screening Tools and Data

Two different tools were utilised to collect data on cognitive function (Gómez-Olivé et al., 2018; Kobayashi et al., 2019; Farrell et al., 2020). The first tool was adapted from the US HRS cognition screening tool. It was translated into the local language, Shangaan, and then back-translated to ensure cultural appropriateness and transferability (Kobayashi et al., 2019; Farrell et al., 2020). It included questions assessing the following measures: orientation, immediate and delayed word recall, numeracy, self-rated memory, and literacy (Gómez-Olivé et al., 2018; Kobayashi et al., 2019; Farrell et al., 2020). A score from 0 to 24, defined here as, ‘total cognition score’, representing global cognition, was used as one outcome variable for this study. The OCS-Plus was developed and then validated in a subset of HAALSI participants to capture cognitive domain-specific data without literacy or numeracy bias (Humphreys et al., 2017). Episodic memory, language, executive function, and visuospatial cognition data were collected and transformed into z-scores for each individual per domain thus providing standardised domain-specific scores for this population using methods described by Seidlecki et al. (2008) (Farrell et al., 2020). The sample size was larger for total cognition score than for the domain outcomes.

### DNA Extraction and *APOE* Genotyping

DNA was extracted from buffy coat samples using the QIAsymphony SP (QIAGEN GmbH, QIAGEN Strasse 1, 40,724 Hilden, Germany) at the Sydney Brenner Institute for Molecular Bioscience (SBIMB). TaqMan^™^ single-nucleotide polymorphism (SNP) genotyping was performed using DNA from 1952 individuals for rs429358 and rs7412, of which 1776 were successfully genotyped at both loci. Genotyping was performed on the QuantStudio^™^ 5 Real-Time PCR System (Applied Biosystems, Thermo Fisher Scientific, Carlsbad, United States), and results were analysed using the QuantStudio^™^ Design & Analysis Software. *APOE* alleles were determined by using the genotypes for the SNPs rs429358 (T, C) and rs7412 (C, T). When the combined haplotype was TT, this represented the ε2 allele. When the haplotype was TC ε3, and when the haplotype was CC this was interpreted as ε4.

SNP data for 2,504 individuals from the 1000 Genomes Project (Phase 3) were downloaded from the Ensembl genome browser for the Genome Reference Consortium Human Genome build 37 (GRCh37; ftp://ftp.1000genomes.ebi.ac.uk/vol1/ftp/phase3/data; Accessed 11 Feb, 2021; Auton et al., 2015; 1,000 Genomes Project and AWS, RRID:SCR_008801). Samples were divided according to super population codes as representative of European (EUR), African (AFR), East Asian (EAS), South Asian (SAS), and Admixed American (AMR) ancestries (Auton et al., 2015; 1,000 Genomes Project and AWS, RRID:SCR_008801). *APOE* alleles were determined as above, and frequencies for each of these super populations were compared to our data.

### Statistical Analysis

Allele and genotype frequencies were calculated, and Hardy-Weinberg equilibrium (HWE) was tested using the Pearson’s Chi-squared test. Chi-squared tests were used to test for differences in *APOE* allele distribution and categorical measures of age, sex, level of education, HIV status, each of the 1,000 Genomes super populations and data from four previous African studies (Willis et al., 2003; Gureje et al., 2006; Chen et al., 2010 and Joska et al., 2010). The allele frequency data are represented as pie diagrams on the backdrop of a map of Africa created by MapChart^1^ (2021). In order to assess correlations between *APOE* distribution and age, age was categorised firstly as 10-year intervals, and then as younger (40–59years) vs. older (60years and older). Descriptive statistics was used to summarise continuous (age, total cognition score and domain-specific z-scores) and categorical variables (HIV status, level of education). Two-sample hypothesis testing was used to further tease out the observed association between *APOE* and HIV. This was followed by logistic regression to determine the effects of each allele on HIV status while adjusting for the presence or absence of one of the other alleles. Cognitive function, either as total cognition score or as one of the four above-mentioned domains, were used as outcome variables to test for associations. Linear regression models for each cognitive outcome were tested. Four models were tested as follows: (1) regression using six known genotypes (2) regression comparing 𝜀3 homozygotes to 𝜀4-carriers (3) regression comparing 𝜀4 homozygotes to the rest of the genotypes and (4) regression comparing 𝜀2 homozygotes to 𝜀4 homozygotes. Chi-squared tests, two-sample proportion testing and logistic regression analysis reporting McFadden’s R^2^ were performed using STATA 15.0.585 (StataCorp, 2017; Stata Statistical Software: Release 15, College Station, TX: StataCorp LLC; Stata, RRID:SCR_012763), and summary statistics, regression analyses and correction for multiple testing were performed using R (R Core Team, 2020. R: A language and environment for statistical computing; R Foundation for Statistical Computing, Vienna, Austria. https://www.R-project.org/; R Project for Statistical Computing, RRID:SCR_001905). Bonferroni correction was applied for multiple testing.

## Results

The phenotypic characteristics and covariates of the study population along with the number of participants (n) with data for each variable are shown in Table 1. The mean age was 56.19years, and there were more female participants (58.22%). Education levels in the community were low with the majority having no formal education or having only attended primary school (74.66%). HIV infection was high with 28.48% of the study population living with HIV. Total cognition score was normally distributed with a mean of 11.98. The four standardised domain scores showed considerable variability. The range was particularly wide for visuospatial cognition (−10.627 to 4.771) and executive function (−4.687 to 4.328).

**Table 1:** Summary characteristics of study population.

Measure	*n*	Range	Mean or percentage	*SD*
Age (years)	1776	40 to 80	56.19	10.36
Sex	1776			
Female			58.22%	
Male			41.78%	
Level of education	1772			
No formal education			36.00%	
Primary			38.66%	
High			21.11%	
Tertiary			4.24%	
HIV status	1773			
Negative			71.52%	
Positive			28.48%	
Cognitive measures				
Total cognition score	1776	0 to 24	11.98	4.44
Cognitive domains	1502			
Executive function		−4.687 to 4.328		
Episodic memory		−3.824 to 1.456		
Language		−2.533 to 1.279		
Visuospatial cognition		−10.627 to 4.771		

*Sample number (n) differs per covariate/measure due to data missingness. Z-scores for cognitive domain data are represented only as ranges. Means and standard deviation (SD) are shown for continuous variables and percentages for categorical ones*.

**Figure 1:**
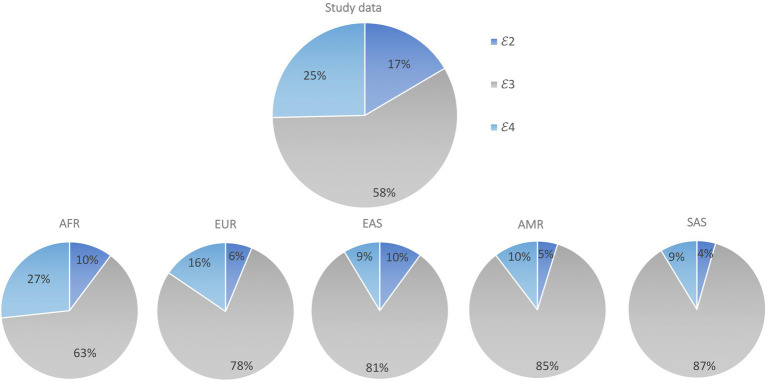
Comparison of *APOE* allele distribution in our data set (Study data) compared to 1,000 Genomes super population data sets AFR, EUR, EAS, SAS and AMR. Pearson’s Chi-squared test was used to assess differences in *APOE* allele frequency between sample populations from each of the 1,000 Genomes super populations and our study sample.

**Figure 2:**
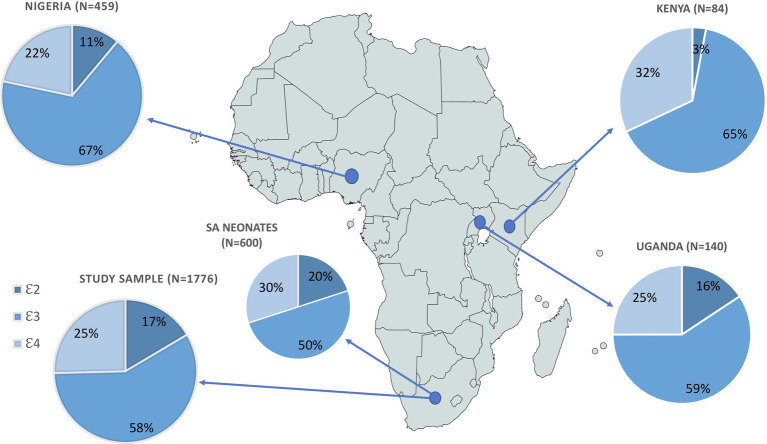
Comparison of *APOE* allele frequency distributions from four African studies, showing sample size for each study. Pearson’s Chi-squared test was used to assess differences in APOE allele frequency between sample populations from each of the African studies referenced by the country of origin/description: Uganda: Willis et al., 2003; Nigeria: Gureje et al., 2006; Kenya: Chen et al., 2010; SA neonates: Joska et al., 2010.

In total, 1776 individuals were successfully genotyped, and all six genotypes were observed with 𝜀2 homozygotes having the lowest frequency (2.9%) and 𝜀3/𝜀3 the highest (34.9%; Table 2). The genotype distribution was in Hardy Weinberg equilibrium (*p*=0.28). Our sample had a similar allele frequency distribution to that of the AFR super population group of Phase 3 of the 1,000 Genomes Project (χ^2^=1.0983, df=2, *p*=0.35; Figure 1). The frequencies of 𝜀2 and 𝜀4 were higher in our sample than in the other populations (EUR, EAS, SAS and AMR representing South American ethnicities). When compared to other African studies (Figure 2), a significant difference in allele frequency distribution was observed between our data and that of Chen et al. (2010) in a study from Kenya (χ^2^=11.058, df=2, *p*=0.004), but due to the small sample size, this difference may not be true.

**Table 2:** *APOE* allele and genotype frequencies.

Allele/Genotype	Number (Frequency)
ℇ2	587 (0.165)
ℇ3	2064 (0.581)
ℇ4	901 (0.254)
**Total**	**3552 alleles**
ℇ2/ℇ2	51 (0.029)
ℇ2/ℇ3	330 (0.186)
ℇ2/ℇ4	155 (0.087)
ℇ3/ℇ3	619 (0.349)
ℇ3/ℇ4	496 (0.279)
ℇ4/ℇ4	125 (0.070)
**Total**	**1776 individuals**

*APOE* allele distribution showed no correlation with sex (χ^2^=1.3329, df=2, *p*=0.514) or level of education (χ^2^=5.7393, df=6, *p*=0.453). No significant difference in allele frequency was observed between age categories per decade nor for younger vs. older individuals (χ^2^=2.0264, df=6, *p*=0.917, and χ^2^=0.4175, df=2, *p*=0.812, respectively). There was evidence of a relationship between HIV status and *APOE* allele frequency (χ^2^=6.9266, df=2, *p*=0.031). Results of hypothesis testing indicated that the proportion of ɛ3 alleles was higher in HIV individuals than in those without HIV (*p*=0.001) and the opposite was observed for ɛ4 (*p*=0.007). Logistic regression results showed the size and direction of the effects of each allele on HIV status, but these associations were not significant as our model had little predictive value (McFadden’s pseudo R^2^=0.0023; Table 3).

**Table 3:** *APOE* allele association with HIV status.

Allele	HIV+(n)	HIV-(n)	OR[95%CI]	*z*	*p*
ℇ2	0.163 (165)	0.166 (421)	0.997 [0.77–1.29]	−0.02	0.980
ℇ3	0.612 (618)	0.569 (1443)	1.149 [0.82–1.60]	0.82	0.412
ℇ4	0.225 (227)	0.265 (672)	0.843 [0.66–1.08]	−1.37	0.172

*Logistic regression was performed to determine size and direction of effects of APOE on HIV status*.

Four multiple regression models were applied, to allow for comparative analyses with other studies, to each of the five cognitive phenotypes. Each model was representative of the comparison between *APOE* genotypes (Model 1: all genotypes; Model 2: 𝜀3 homozygotes vs. 𝜀4-carriers; Model 3: 𝜀4 homozygotes vs. the rest of the genotypes; and Model 4: 𝜀2 homozygotes vs. 𝜀4 homozygotes) and their effect on cognition while adjusting for covariates (age, sex, level of education and HIV status). Age and level of education were significantly associated with cognitive function in all of the models, and sex was significant for executive function, language and visuospatial cognition for models 1–3 where being male increased the standard estimates for each domain (Table 4 and Supplementary Table S1). Significant associations were observed in Model 1 (Table 4) for *APOE* genotypes, where 𝜀2/𝜀2 was associated with higher levels of executive function by approximately half a standard deviation (*β*=0.489, SE=0.57, *p*=0.037) and lower levels (but not significantly) when participants were heterozygous 𝜀2-carriers, and 𝜀3/𝜀4 was associated with lower levels of visuospatial cognition (*β*=−0.286, SE=0.14, *p*=0.046). These effects (f^2^=0.395 and f^2^=0.597, respectively) were not significant after Bonferroni correction for multiple testing (*p*=0.337 and 0.416, respectively).

**Table 4:** Linear regression model 1 using all genotypes to estimate the effects of *APOE* and covariates on cognitive function.

	Total cognition		Executive function		Language		Memory		Visuospatial cognition	
Model	*Coefficient*	*SE*	*Coefficient*	*SE*	*Coefficient*	*SE*	*Coefficient*	*SE*	*Coefficient*	*SE*
All genotypes										
ℇ3/ℇ4	−0.306	0.24	−0.042	0.10	−0.048	0.04	−0.062	0.06	−0.286^*^	0.14
ℇ3/ℇ2	−0.134	0.27	0.024	0.11	0.013	0.04	0.021	0.07	−0.063	0.16
ℇ2/ℇ4	−0.107	0.35	−0.025	0.15	−0.015	0.05	0.066	0.09	−0.060	0.22
ℇ4/ℇ4	0.114	0.39	−0.066	0.16	−0.058	0.06	−0.036	0.09	−0.164	0.24
ℇ2/ℇ2	0.434	0.57	0.489^*^	0.23	0.155	0.09	0.099	0.14	0.470	0.35
Age	−0.093^***^	0.01	−0.037^***^	<0.01	−0.013^***^	<0.01	−0.029^***^	<0.01	−0.081^***^	0.01
Male	−0.018	0.19	0.264^*^	0.08	0.059^*^	0.03	−0.05	0.05	0.383^*^	0.12
Education	1.758^***^	0.12	0.823^***^	0.05	0.355^***^	0.02	0.349^***^	0.03	1.394^***^	0.07
HIV positive	0.017	0.21	−0.004	0.09	−0.025	0.03	−0.041	0.05	−0.175	0.13
Adjusted R-squared	0.221	3.91	0.283	1.48	0.325	0.54	0.245	0.87	0.374	2.20

*Standardised coefficients represent estimated changes in cognitive function. Domain estimates (executive function, language, memory, visuospatial cognition) change in standard deviation units. Adjusted R-squared values per model with residual standard error (SE) is shown*.

* *p<0.05*;

^**^p<0.00001;

*** *p<2e-16*.

## Discussion

This cross-sectional study assessed the potential effect of *APOE* genetic variation on five measures of cognition in a rural-dwelling SA population. Participants were adults of African ethnicity aged 40years and older. We aimed to address the lack of African data for large cohorts that can be used to examine the genetics of cognition and cognitive decline, specifically in rural areas where vulnerability to cognitive impairment and dementia is becoming a major public health concern (Kobayashi et al., 2019). Association studies have observed genetic variation in neurocognitive traits between different ethnicities (Savitz et al., 2006; Fitzgerald et al., 2020). Here, we determined *APOE* allele frequency distribution for our dataset and then compared this to existing data from other population groups. The genetic contribution to cognitive function is complex, and the tests used to define levels of cognition have made equitable comparisons between studies difficult (Savitz et al., 2006; Goriounova and Mansvelder, 2019; Fitzgerald et al., 2020). This is especially challenging in a low literacy setting despite the development of tests to assess cognition regardless of levels of literacy. Nonetheless, both literacy and level of education are still associated with cognition in this study cohort (Kobayashi et al., 2019; Farrell et al., 2020). Traditionally, intelligence has been measured using the intelligence quotient (IQ) or Spearman’s *g* (used in meta-analyses), which are biased towards access to formal education (as they focus mostly on aspects related to literacy and numeracy) (Goriounova and Mansvelder, 2019; Fitzgerald et al., 2020). Here, we focused on latent cognitive ability which encompasses domain-specific and global cognitive function outside of educational attainment (Goriounova and Mansvelder, 2019; Fitzgerald et al., 2020). Collecting cognition data is further complicated by using screening tools which have not been validated for transferability across populations; therefore, the OCS-Plus tool was developed to provide an alternative method for capturing cognition data in low-income populations that was neither reliant on literacy nor numeracy (Humphreys et al., 2017). The HAALSI baseline study provided an appropriate setting to validate this tablet-based tool, providing population-specific cognitive function data for an understudied and socially homogeneous population (Humphreys et al., 2017; Gómez-Olivé et al., 2018). Leveraging these data and further capitalising on the overlap of AWI-Gen participants for which DNA was available, we sought to explore genetic effects of *APOE* on cognitive function.

*APOE* genotyping revealed that ɛ2 and ɛ4 are generally more common in African populations, including in our own, with SA and Uganda having the highest frequencies of ɛ2 among African populations (Figure 2). Although these allele frequencies differed significantly from the Chen et al. (2010) cross-sectional study of a population in Kenya, the small sample size of this Kenyan study was insufficient for accurate comparison of intra-continental variation of *APOE*. The higher ɛ4 frequency in Africans has been attributed to selective advantage as protection against infectious disease (Corbo and Scacchp, 1999; Fujioka et al., 2014; Van Exel et al., 2017; Smith et al., 2019). *APOE* ɛ4 has been suggested to be protective against hepatitis C infection as well as carriers being observed to have a better prognosis post infection (Kuhlmann et al., 2010; Mueller et al., 2016; Smith et al., 2019). There is also evidence that ɛ4 inhibits growth of *Plasmodium falciparum* conveying a protective effect against malaria (Fujioka et al., 2014; Van Exel et al., 2017; Smith et al., 2019). Higher observed ɛ4 frequency in Africans has also been attributed to the hypothesis that it is beneficial for fertility and early infant survival (Joska et al., 2010; Van Exel et al., 2017; Smith et al., 2019). Despite these proposed early life advantages, it has also been associated with increased morbidity and mortality later in life (Hendrie et al., 1995; Savitz et al., 2006; Burt et al., 2008; Chang et al., 2011; Panos et al., 2013; Kotze et al., 2015; Van Exel et al., 2017; O’Donoghue et al., 2018; Smith et al., 2019; Kuo et al., 2020). Smaller African studies mainly assessing *APOE* association with AD showed no significant associations (Willis et al., 2003; Gureje et al., 2006; Chen et al., 2010; Joska et al., 2010). The genetic associations between ɛ4 and neurocognitive phenotypes (mostly AD due to its highly replicated association with increased susceptibility) have observed weaker effects in those of admixed African ancestry (AA and Brazilians) and lack of association in Africans (Osuntokun et al., 1995; Willis et al., 2003; Small et al., 2004; Wisdom et al., 2011; Hendrie et al., 2015; O’Donoghue et al., 2018; Gouveia et al., 2019).

Previous epidemiological studies on the HAALSI cohort have reported the effects of age, sex, level of education and HIV status on these same cognitive phenotypes (Kobayashi et al., 2019; Asiimwe et al., 2020; Farrell et al., 2020). Higher educational attainment and younger age are known to be associated with better cognitive performance, and we observed these same education-related effects across all cognitive phenotypes in our study and in our regression models. In this study population, we observed better performance in the domains of executive function and language in male participants, a finding that had already been published by Farrell et al. (2020) who attributed this sex difference to socio-cultural differences and access to education (Kobayashi et al., 2019). Other studies have suggested that older women generally have better cognitive performance than their male counterparts possibly due to the effects of oestrogen or differences in cognitive reserve (Wu et al., 2012; Hayat et al., 2014; Clifford et al., 2015; Sundermann et al., 2016; Lipnicki et al., 2019). Sex effects appear to be domain-specific, and this has been observed in studies where women performed better in tests assessing memory than men irrespective of educational background (Clifford et al., 2015; Sundermann et al., 2016).

For this study, we used HIV status as a covariate because of the high prevalence of HIV and its known contribution to cognitive impairment through HAND (Andres, 2011; Panos et al., 2013; Chang et al., 2015; Rebeck, 2017; Geffin and McCarthy, 2018; Hulgan et al., 2018). Upon assessment of the *APOE* distributions for each of the covariates, we observed statistical differences between allele distribution according to HIV status. When modelling the effects of each allele on HIV status, logistic regression indicated that our model consisting of HIV status conditional on *APOE* allele was inadequate, which could be due to small sample size. Although the proportion of ɛ3 alleles was significantly higher in HIV positive individuals, the relative effect of this allele on HIV status compared to the effects of the other alleles was not significant. This is contrary to another SA study reporting lower ɛ3 allele frequency in HIV positive adults vs. newborn controls (Joska et al., 2010). The opposite relationship for ɛ4, with a higher proportion observed in those without HIV, was also not significant, but our results suggested that ɛ3 is the risk allele for HIV infection. *APOE* ɛ4 has been linked to AIDS severity and increased mortality especially in ɛ4 homozygotes (Valcour et al., 2004; Burt et al., 2008; Chang et al., 2015; Wendelken et al., 2016). It has been associated with faster disease progression and higher viral load in seropositive individuals due to enhanced entry of the virus into T cells (Burt et al., 2008; Kuhlmann et al., 2010). This may explain why we see a higher proportion of ɛ3 in our HIV positive sample as faster disease progression and poorer prognosis may have resulted in earlier HIV-related mortality in ɛ4-carriers. The mortality profile of the community from which this cohort was recruited was subject to high levels of HIV-associated death, and increased all cause death in age groups from 15 to 64years in the years preceding data collection for this study (Kabudula et al., 2014). HIV progression is accompanied by domain-specific cognitive deficits characterised as HAND, where ɛ4 is associated with reduced performance in these domains (Chang et al., 2011; Panos et al., 2013; Chang et al., 2015; Wendelken et al., 2016; Geffin and McCarthy, 2018). Some studies have failed to replicate these findings and have reported either no association between ɛ4 and HIV, or lack of association between ɛ4 and HAND (Joska et al., 2010; Morgan et al., 2013; Becker et al., 2015; Cysique et al., 2015). HIV status had no significant effect on cognitive function in any of our models. This result may be attributed to widespread use of antiretroviral therapy in this community and also an indication that people living with HIV may have better access to healthcare and thus are better able to manage both age-related and HIV-associated morbidity (Kabudula et al., 2014; Asiimwe et al., 2020; Rosenberg et al., 2020). There is evidence that this sample population may have a high prevalence of HAND within the HIV positive group, but further evidence for trends in the development of accurately diagnosed HAND and seropositivity in the context of *APOE* have not been explored (Asiimwe et al., 2020; Rosenberg et al., 2020).

Although we observed possible associations of *APOE* genetic variants with executive function and visuospatial cognition, these associations did not survive correction for multiple testing. *APOE* ɛ2 homozygosity was linked to increased executive function scores and ɛ3/ɛ4 heterozygosity to lower visuospatial performance. Other studies comparing performance on cognition tests between ɛ3 homozygotes and ɛ4-carriers observed impaired executive function, episodic memory, global cognition and processing speed in ɛ4-carriers which was exacerbated in ɛ4 homozygotes (Lipnicki et al., 2017; O’Donoghue et al., 2018; Hays et al., 2019). Replication of these findings has proven challenging (Savitz et al., 2006; O’Donoghue et al., 2018; Fitzgerald et al., 2020). We observed an increase of ~0.5 standard deviations in the domain of executive function for ɛ2/ɛ2 individuals and~0.3 standard deviations reduction in visuospatial ability of ɛ3/ɛ4 individuals, larger effects than those of a large meta-analysis by Small et al. (2004). They observed reduced performance of <0.1 standard deviations for global cognition, episodic memory and executive function in ɛ4 carriers, with significantly poorer cognitive performance associated with ɛ4 homozygosity (Small et al., 2004). A later meta-analysis of 77 studies totalling 40,942 individuals observed similar results when assessing the effects of *APOE* on normal cognitive function (Wisdom et al., 2011). Contrary to the findings of Wisdom et al. (2011), we did not observe significantly higher memory scores in ɛ2 homozygotes compared to ɛ4 homozygotes.

We report on baseline genetic and domain-specific cognitive function data for a large African study cohort. However, sample size was a limitation for observing small genetic effects for associations between *APOE* genotypes and cognitive function. Meta-analyses have estimated the effect sizes of *APOE* on different cognitive domains to vary between approximately 0.07 and 0.002 (Small et al., 2004; Wisdom et al., 2011). But to detect the effect size of 0.01 estimated by Wisdom et al. (2011) for visuospatial ability, we would need to double our sample size. Our models explained modest variation of cognitive function mostly due to highly significant associations observed with age (cognition score decreasing with increasing age) and level of education (cognition score increasing as higher levels of education are achieved), which are known to influence normal cognitive function. It has been suggested that cognition changes throughout the lifespan and that ɛ4-carriers perform better at cognition tests when they are younger due to antagonistic pleiotropy and the compensation of other pathways which improve cognition and cognitive reserve at younger ages, but become detrimental over the age of 60years (Chang et al., 2011; Panos et al., 2013; Rebeck, 2017; O’Donoghue et al., 2018). We were unable to confirm this in our study, but longitudinal data from HAALSI’s second wave of data collection will enable the assessment of the effects of *APOE* on age-related cognitive decline.

There is little data on cognitive function and domain-specific genetic associations in African populations. Research in European and Asian populations has shown that *APOE* genetic variation is associated with cognition and differences in susceptibility to several diseases. Despite previous associations between ɛ4 and cognition, we were unable to replicate these findings. Instead, we observed a trend towards higher executive function in ɛ2 homozygotes and lower visuospatial cognition in ɛ3/ɛ4 heterozygotes. These associations were strongly moderated by education and age, and there was minimal indication for the independent influence of *APOE* on latent cognitive function. Cognition screening tools which are culturally adapted and unbiased in terms of literacy and numeracy are essential for the accurate interpretation of cognition in populations with low levels of education. The inconclusive results of this study may further catalyse African exploratory studies of the complex genetics behind cognition, and the role of known environmental confounders that are becoming increasingly important in the prediction, prevention, and treatment of dementia in low- to middle-income countries.

## Data Availability Statement

The HAALSI baseline data are publicly available at the Harvard Center for Population and Development Studies (HCPDS) programme website [www.haalsi.org]. Data are also accessible through the MRC/Wits Agincourt Research Unit’s data repository [https://data.agincourt.co.za/index.php/catalog/18], the Inter-university Consortium for Political and Social Research (ICPSR) at the University of Michigan [www.icpsr.umich.edu], and the INDEPTH Data Repository [https://www.indepth-ishare.org/index.php/catalog/113]. The linked genetic and phenotypic data is available through the Harvard Center for Population and Development Studies (HCPDS) programme website [https://dataverse.harvard.edu/dataset.xhtml?persistentId=doi:10.7910/DVN/QWSQXR]. Requests for these data through the website are made to the principal investigators of the HAALSI study.

## Ethics Statement

The studies involving human participants were reviewed and approved by University of the Witwatersrand, Johannesburg, Human Research Ethics Committee [Wits HREC (Medical)] (M141159, M121029, and M170916), the Harvard T.H. Chan School of Public Health, Office of Human Research Administration (C13-1608-02), and the Mpumalanga Provincial Research and Ethics Committee (approved: 2014/10/22). The patients/participants provided their written informed consent to participate in this study.

## Author Contributions

The study was developed by CS together with MR, AN, ST and LB. MF transformed and analysed the raw cognition data and provided information on cognitive phenotypes. CS performed the analyses and drafted the manuscript, and all other authors critically revised and approved the manuscript.

## Funding

The HAALSI study was funded by the National Institute on Aging (P01 AG041710) and is nested within the Agincourt Health and Demographic Surveillance System site, supported by the University of the Witwatersrand and Medical Research Council, South Africa, and the Wellcome Trust, United Kingdom (grants no. 058893/Z/99/A; 069683/Z/02/Z; 085477/Z/08/Z; and 085477/B/08/Z). It has been carried out through a collaboration between the Harvard Center for Population and Development Studies from Harvard T.H. Chan School of Public Health, MRC/Wits Rural Public Health and Health Transitions Research Unit from School of Public Health at the University of the Witwatersrand in South Africa, and the INDEPTH Network in Accra, Ghana. The content is solely the responsibility of the authors and does not necessarily represent the official views of National Institute on Aging or the Wellcome Trust.

The AWI-Gen Study was funded by the National Human Genome Research Institute (NHGRI), Office of the Director (OD), Eunice Kennedy Shriver National Institute Of Child Health & Human Development (NICHD), the National Institute of Environmental Health Sciences (NIEHS), the Office of AIDS research (OAR) and the National Institute of Diabetes and Digestive and Kidney Diseases (NIDDK), of the National Institutes of Health (NIH) under award number U54HG006938 and its supplements, as part of the H3Africa Consortium. The study design, conclusions, and opinions expressed in this paper do not necessarily represent the official views of the National Institutes of Health.

CS was supported by the NIH grant U54HG006938. CS was funded by the National Research Foundation (NRF) Thuthuka funding instrument grant no. TTK160602167377, and the NIH Fogarty International Centre SEED funding (D43TW008330) under the umbrella of the Wits Non-Communicable Disease Research Leadership Program. The views expressed herein are those of the authors and do not necessarily reflect those of the NIH, the NRF, or the NIH Fogarty International Centre.

## Conflict of Interest

The authors declare that the research was conducted in the absence of any commercial or financial relationships that could be construed as a potential conflict of interest.

## Publisher’s Note

All claims expressed in this article are solely those of the authors and do not necessarily represent those of their affiliated organizations, or those of the publisher, the editors and the reviewers. Any product that may be evaluated in this article, or claim that may be made by its manufacturer, is not guaranteed or endorsed by the publisher.

## Footnotes

^1^ https://mapchart.net/africa.html

## Abbreviations

AA: African American
AD: Alzheimer’s Disease
*APOE*: Apolipoprotein E Gene
AFR: 1,000 Genomes African super population group
AMR: 1,000 Genomes Admixed American super population group
AWI-Gen: Africa Wits-INDEPTH Partnership for Genomic Studies
CVDs: Cardiovascular Diseases
EAS: 1,000 Genomes East Asian super population group
EUR: 1,000 Genomes European super population group
GRCh37: Genome Reference Consortium Human genome build 37
HAALSI: The Health and Aging in Africa: A Longitudinal Study of an INDEPTH Community in South Africa
HAND: HIV-Associated Neurocognitive Disorder
HIV: Human Immunodeficiency Virus
HWE: Hardy Weinberg Equilibrium
LOAD: Late-onset Alzheimer’s Disease
MCI: Mild Cognitive Impairment
OCS-Plus: Oxford Cognition Screen Plus
SAS: 1,000 Genomes South Asian super population group
SBIMB: Sydney Brenner Institute for Molecular Bioscience
SNPs: Single-nucleotide polymorphisms
US HRS: United States Health and Retirement Study
