# Corrigendum: Apolipoprotein E Genetic Variation and its Association With Cognitive Function in Rural-Dwelling Older South Africans

**DOI:** 10.3389/fgene.2022.795349

**Published:** 2022-04-20

**Authors:** Cassandra C. Soo, Meagan T. Farrell, Stephen Tollman, Lisa Berkman, Almut Nebel, Michèle Ramsay

**Affiliations:** ^1^ Sydney Brenner Institute for Molecular Bioscience, Faculty of Health Sciences, University of the Witwatersrand, Johannesburg, South Africa; ^2^ Division of Human Genetics, National Health Laboratory Service and School of Pathology, Faculty of Health Sciences, University of the Witwatersrand, Johannesburg, South Africa; ^3^ Harvard Center for Population and Development Studies, Harvard University, Cambridge, MA, United States; ^4^ MRC/Wits Rural Public Health and Health Transitions Research Unit, School of Public Health, Faculty of Health Sciences, University of the Witwatersrand, Johannesburg, South Africa; ^5^ Department of Social and Behavioral Sciences, Harvard T.H. Chan School of Public Health, Boston, MA, United States; ^6^ Institute of Clinical Molecular Biology, Kiel University, Kiel, Germany

**Keywords:** apolipoprotein E ε4, cognitive function, african population, educational attainment, memory, executive function, visuospatial ability, language

In the original article, there was a mistake in the legend for **Table 4** as published. There was a typo in the legend: “Standardised coefficients represent estimated changes in cognitive function. Domain estimates (executive function, language, memory, visuospatial cognition) change in standard deviation units. Adjusted R-squared values per model with residual standard error (SE) is shown. *p < 0.5, **p < 0.00001, ***p < 2e-16*.*” The correct legend is “Standardised coefficients represent estimated changes in cognitive function. Domain estimates (executive function, language, memory, visuospatial cognition) change in standard deviation units. Adjusted R-squared values per model with residual standard error (SE) is shown. *p < 0.05, **p < 0.00001, ***p < 2e-16*.*”

In the original article, there was a mistake in the legend for **Supplementary Table S1** as published. There was a typo in the legend: “Standardised coefficients represent estimated changes in cognitive function. Domain estimates (executive function, language, memory, visuospatial cognition) change in standard deviation units. Adjusted R-squared values per model with residual standard error (SE) is shown. *p < 0.5, **p < 0.00001, ***p < 2e-16*.*” The correct legend is “Standardised coefficients represent estimated changes in cognitive function. Domain estimates (executive function, language, memory, visuospatial cognition) change in standard deviation units. Adjusted R-squared values per model with residual standard error (SE) is shown. *p < 0.05, **p < 0.00001, ***p < 2e-16*.*”

The authors apologize for this error and state that this does not change the scientific conclusions of the article in any way. The original article has been updated.

